# PSA Depletion Induces the Differentiation of Immature Neurons in the Piriform Cortex of Adult Mice

**DOI:** 10.3390/ijms22115733

**Published:** 2021-05-27

**Authors:** Simona Coviello, Bruno Benedetti, Dominika Jakubecova, Maria Belles, Patrycja Klimczak, Yaiza Gramuntell, Sebastien Couillard-Despres, Juan Nacher

**Affiliations:** 1 Neurobiology Unit, Program in Neurosciences and Institute of Biotechnology and Biomedicine (BIOTECMED), Universitat de València, 46100 Burjassot, Spain; simona.coviello@uv.es (S.C.); maria.belles@uv.es (M.B.); patrycja.a.klimczak@uv.es (P.K.); yaigra@alumni.uv.es (Y.G.); 2 Spinal Cord Injury and Tissue Regeneration Center Salzburg (SCI-TReCS), Institute of Experimental Neuroregeneration, Paracelsus Medical University, 5020 Salzburg, Austria; bruno.benedetti@pmu.ac.at (B.B.); d.jakubecova@pmu.ac.at (D.J.); 3 Austrian Cluster for Tissue Regeneration, 1200 Vienna, Austria; 4 Spanish National Network for Research in Mental Health (CIBERSAM), 28029 Madrid, Spain; 5 Fundación Investigación Hospital Clínico de Valencia, INCLIVA, 46010 Valencia, Spain

**Keywords:** PSA-NCAM, neuronal maturation, neuronal precursors, olfactory cortex, doublecortin

## Abstract

Immature neurons are maintained in cortical regions of the adult mammalian brain. In rodents, many of these immature neurons can be identified in the piriform cortex based on their high expression of early neuronal markers, such as doublecortin (DCX) and the polysialylated form of the neural cell adhesion molecule (PSA-NCAM). This molecule plays critical roles in different neurodevelopmental events. Taking advantage of a DCX-CreERT2/Flox-EGFP reporter mice, we investigated the impact of targeted PSA enzymatic depletion in the piriform cortex on the fate of immature neurons. We report here that the removal of PSA accelerated the final development of immature neurons. This was revealed by a higher frequency of NeuN expression, an increase in the number of cells carrying an axon initial segment (AIS), and an increase in the number of dendrites and dendritic spines on the immature neurons. Taken together, our results demonstrated the crucial role of the PSA moiety in the protracted development of immature neurons residing outside of the neurogenic niches. More studies will be required to understand the intrinsic and extrinsic factors affecting PSA-NCAM expression to understand how the brain regulates the incorporation of these immature neurons to the established neuronal circuits of the adult brain.

## 1. Introduction

New neurons are generated in the canonical neurogenic sites of the adult mammalian brain: the subventricular zone (SVZ) [1] and the subgranular zone (SGZ) of the hippocampal dentate gyrus [2]. These cells are transiently immature, and many of them incorporate into the circuitry. Interestingly, different studies over the past 20 years have found additional reservoirs of immature neurons in the adult mammalian brain, the so-named noncanonical neurogenic niches [3]. In rodents, many of these immature neurons are located in the layer II of the piriform cortex (PCX) [4,5,6]. By contrast, mammals with more complex cerebral cortices, including humans, have more widespread distribution of these neurons throughout the neocortex [7,8,9,10,11,12].

Immature neurons in the PCX layer II are generated during embryonic development [6,9,13] and seem to disappear progressively with aging [14] due to their maturation and incorporation into local cortical networks as mature glutamatergic neurons [11]. The immature neurons in the PCX layer II can be classified into different subtypes depending on their morphology, physiological properties and degree of maturation [13], namely: (1) tangled cells, which are very small and have very short and intricate processes; (2) complex cells, which are more mature and display axon initial segments (AIS) and typical apical dendrites in layer I [15]. The maturation of these tangled and complex cells is accompanied by increased the complexity of their dendritic arbor and the density of dendritic spines, which are associated with presynaptic markers, such as synaptophysin (SYN) and the vesicular glutamate transporter-1 (VGLUT1) [11,15]. Moreover, large complex cells have puncta expressing SYN and the vesicular GABA transporter (VGAT), an inhibitory synaptic marker, in their perisomatic region [11] suggesting increased their connectivity with the surrounding network. The latter could be functionally confirmed by the increasing frequency of postsynaptic currents (PSCs) measured on the complex cells upon maturation [15].

Immature neuronal markers, such as the polysialylated form of the neural cell adhesion molecule (PSA-NCAM) and the cytoskeleton-associated protein doublecortin (DCX), are widely used to identify this population of immature cells [16,17,18,19,20]. The expression of these markers is transient since immature neurons down-regulate them progressively as they undergo maturation [11,14]. The prominent role of PSA-NCAM in structural remodeling and its involvement in several neurodevelopmental events, such as neuronal migration, neurite outgrowth, or synaptogenesis, has been extensively investigated [21,22,23,24,25]. Furthermore, the enzymatic or genetic manipulation of PSA-NCAM promotes neuronal plasticity in different contexts [26,27,28,29]. Interestingly, the targeted removal of PSA moiety from NCAM using the specific enzyme Endo-Neuraminidase-N (Endo-N) [30] promotes the differentiation of subventricular [31] and hippocampal [32] neuronal progenitor cells. Hence, the anti-adhesive and insulating properties of PSA-NCAM appear to be crucial for the maturation kinetics of these immature neurons. Yet, the role of PSA-NCAM on the final differentiation of immature neurons residing in noncanonical neurogenic niches, such as the PCX, remains to be elucidated.

Therefore, this study addresses the involvement of PSA-NCAM in the final stages of the development of immature neurons in the PCX layer II using a tamoxifen-inducible transgenic mouse line with Cre recombinase under the control of the DCX promoter (DCX-CreERT2/Flox-EGFP) [33]. In this system, we could trace the maturation of complex cells following a unilateral intracortical injection of Endo-N, removing PSA from NCAM in the PCX of one hemisphere.

## 2. Results

### 2.1. Endo-N Efficiently Depleted PSA from the PCX

**Figure 1:**
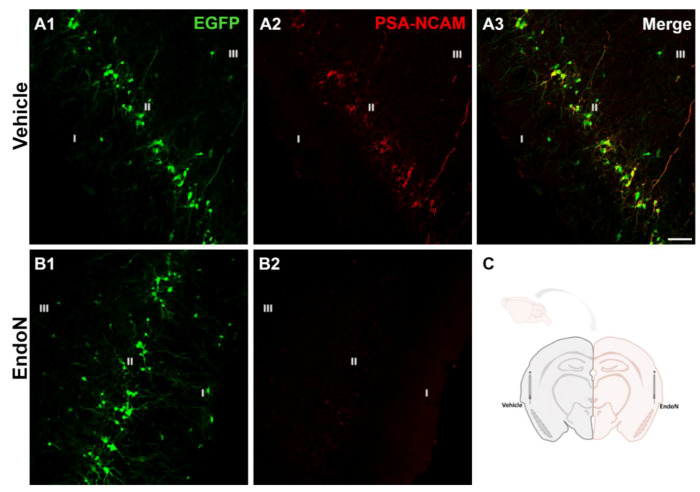
Removal of PSA in the PCX by Endo-N. (**A**,**B**) Confocal microscopic images of EGFP (green) and PSA-NCAM (red) immunoreactive neurons in the vehicle (**A1**–**3**) and Endo-N (**B1**,**2**) injected hemispheres. Note the complete absence of PSA-NCAM expression in the hemisphere injected with Endo-N. (**C**) Drawing showing the hemispheres injected with vehicle (left) or Endo-N (right). Scale bar: 70 µm.

Two weeks after the injection of Endo-N in the ectorhinal cortex of the right hemisphere, PSA was completely depleted in the PCX from −1.58 mm to 2.54 mm Bregma coordinates [34] (Figure 1).

### 2.2. Classification of EGFP^+^ Neurons and Impact of PSA Depletion in the PCX Layer II

**Figure 2:**
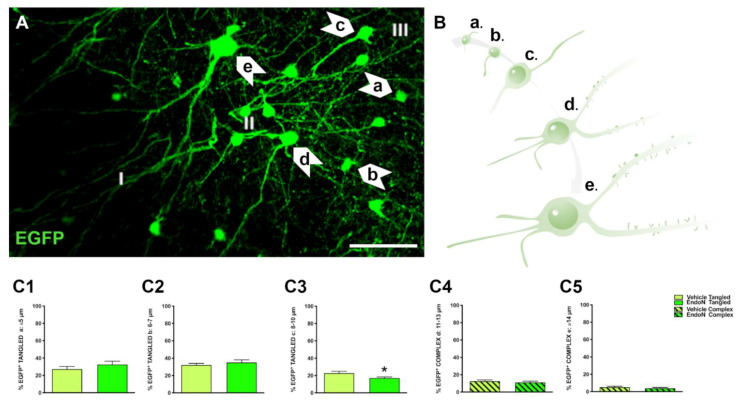
Classification of EGFP^+^ neurons and impact of PSA depletion. (**A**) Single confocal section showing the distribution of EGFP^+^ cells in the PCX layer II. Note the different subtypes of cells indicated by arrows. Scale bar = 50 µm (**B**) Schematic representation showing the different maturational stages of EGFP^+^ cells: a: ≤ 5 µm; b: 6–7 µm; c: 8–10 µm; d: 11–13 µm; e: ≥ 14 µm (**C1**–**5**) Graphs representing the percentage of the different types and maturational stages of EGFP^+^ neurons in the PCX layer II. Asterisks indicate statistically significant differences between hemispheres (Vehicle vs. Endo-N) after paired Student’s *t*-test. (* *p* ≤ 0.05) Scale bar: 100 µm.

In the PCX layer II, EGFP^+^ neurons were classified into tangled and complex cells as described in previous studies [6,11]. In addition, EGFP^+^ neurons were further divided in 5 subgroups according to their soma diameter and maturational stage: tangled a: small tangled cells (ø ≤ 5 µm); tangled b: tangled cells with short dendritic arborizations (ø = 6–7 µm); tangled c: tangled cells with longer dendritic arborizations (ø = 8–10 µm); complex d: larger complex cells with thin basal processes and dendritic spines (ø = 11–13 µm) complex e: mature, complex cells with larger dendrites and mature dendritic spines (ø ≥ 14 µm) (Figure 2A,B). To study the impact of PSA depletion on EGFP^+^ neurons, we analyzed their distribution into the 5 subpopulations (Figure 2C) for the vehicle-treated vs. the Endo-N-treated hemisphere. As previously observed, tangled cells were more abundant than complex cells in both hemispheres. Importantly, a significant decrease in the percentage of tangled cells with 8–10 µm diameter was observed in the hemisphere treated with Endo-N (*p* = 0.02, Figure 2C3).

### 2.3. PSA Depletion Promoted Expression of Mature Neuronal Markers in EGFP^+^ Cells of the PCX Layer II

**Figure 3:**
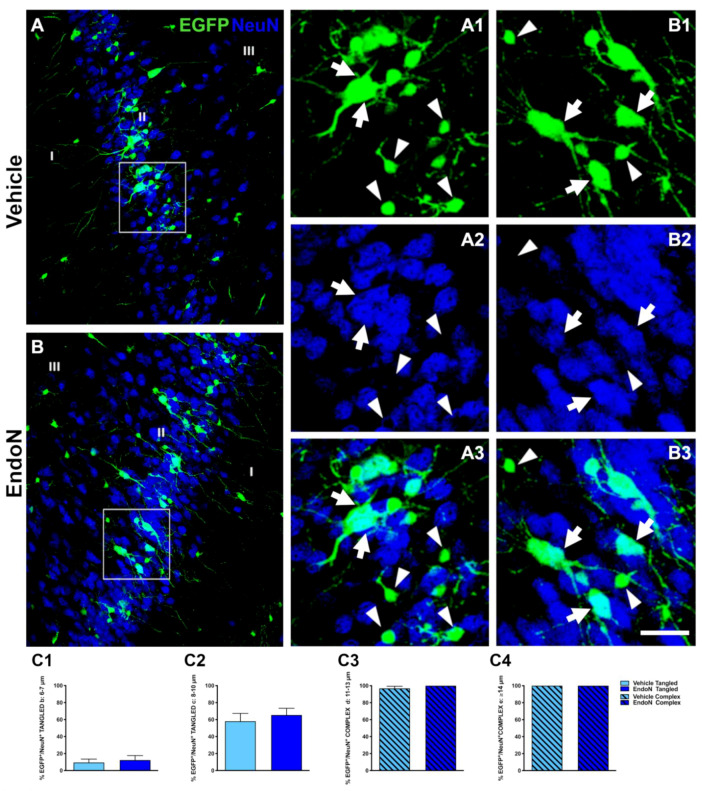
Effects of Endo-N injection on the percentage of EGFP^+^ cells displaying NeuN^+^ nuclei**.** (**A**,**B**) Confocal images showing EGFP^+^ cells (green) and NeuN-expressing cells (blue). (**A1**–**3**,**B1**–**3**) Higher magnifications of the squared areas in A and B. In these insets, tangled and complex EGFP^+^ cells are indicated by arrowheads and arrows, respectively. Note the presence of a larger number of EGFP^+^ complex cells labeled with NeuN in the Endo-N hemisphere. (**C1**–**4**) Graphs representing the percentage of EGFP^+^/NeuN^+^ neurons classified as tangled or complex cells according to their diameter. Scale bar: 70 µm.

To study whether the Endo-N injection could accelerate the final stages of EGFP^+^ cells maturation, we performed immunohistochemical staining against NeuN, a marker of mature neurons [35], and CAMK-II, a protein exclusively found in fully differentiated excitatory neurons [36]. Many EGFP^+^ complex cells displaying NeuN immunoreactive nuclei were found in both hemispheres (Figure 3A,B). We observed that 100% of EGFP^+^ cells from the largest soma group (diameter > 14 µm) expressed NeuN, whereas none of the EGFP^+^ cells with a soma diameter < 5 µm expressed NeuN, regardless of the treatment received. For all 3 remaining subpopulations of EGFP^+^ cells, the percentage of NeuN expression increased upon Endo-N treatment. However, this difference was not significant (Figure 3C3).

In contrast, the EGFP/CAMK-II co-expression analysis did not reveal differences between vehicle and Endo-N-treated hemispheres. It should be noted that CAMK-II expression was not detected in EGFP^+^ cells with ≤5 µm or 6–7 µm diameters.

**Figure 4:**
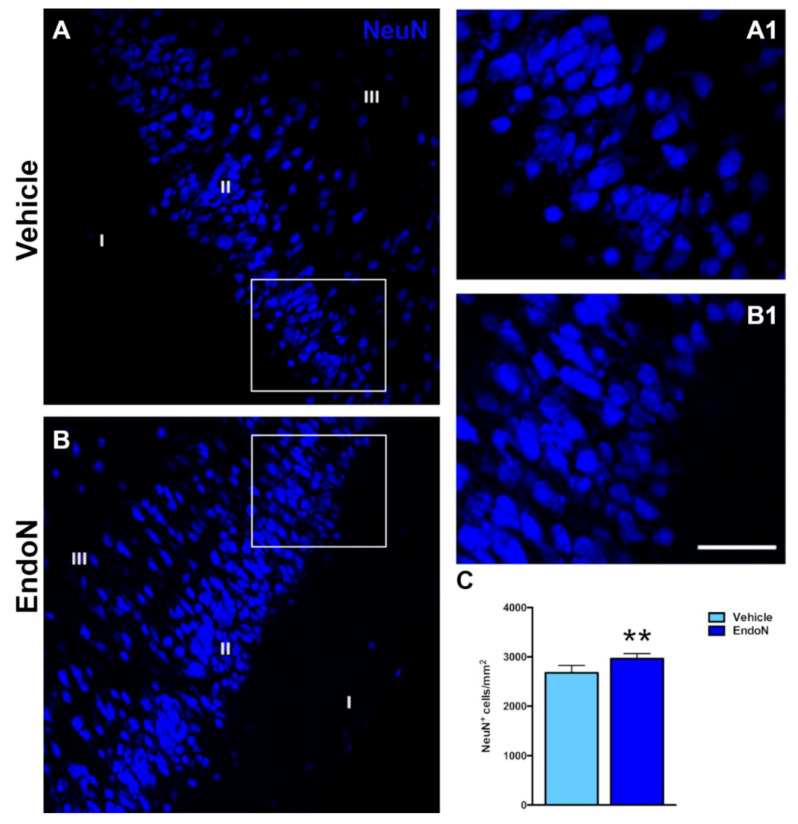
Effects of Endo-N injection on the density of NeuN-expressing cells. (**A**,**B**) Single confocal sections showing the expression of NeuN in the PCX. (**A1**,**B1**) are higher magnifications of the squared areas in (**A**,**B**), which show the different densities of NeuN immunoreactive nuclei in the PCX layer II. (**C**) Graph showing the effect of Endo-N injection on the density of NeuN immunoreactive nuclei in the PCX layer II. Asterisks indicate statistically significant differences between hemispheres (vehicle vs. Endo-N) after paired Student’s *t*-test. (** *p* < 0.01). Scale bar represents 70 µm for (**A**,**B**) and 120 µm for (**A1**,**B1**).

The effects of Endo-N injection on the density of NeuN-expressing and CAMK-II-expressing cells in the PCX layer II were also investigated. Interestingly, the density of NeuN immunoreactive nuclei was significantly higher (*p* = 0.007) in the Endo-N versus the vehicle-treated hemisphere (Figure 4). In contrast, no significant changes were observed in the density of CAMK-II-expressing cells. (Supplementary Materials Figure S1).

### 2.4. PSA Depletion Increased the Density of EGFP^+^ Cells Bearing an Axon Initial Segment in the PCX Layer II

**Figure 5:**
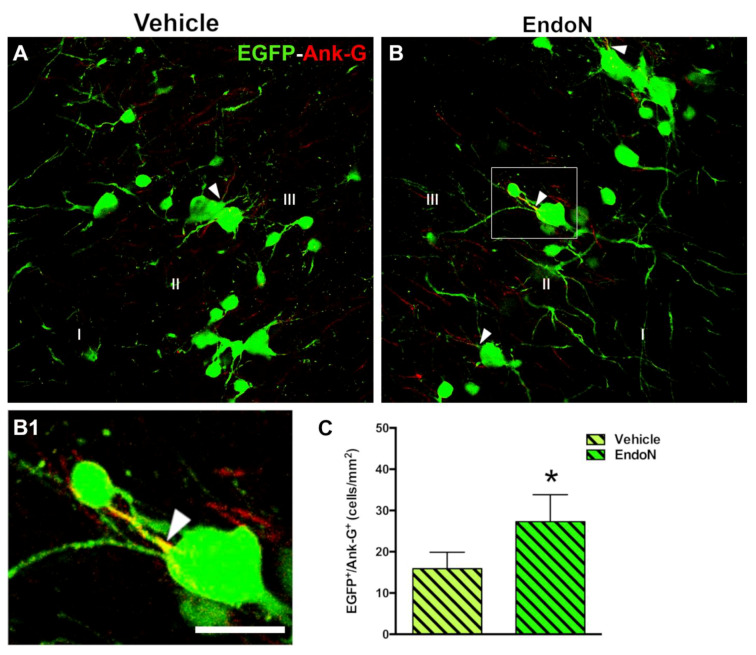
Effects of Endo-N injection on the density of EGFP^+^ complex cells displaying an AIS. (**A**,**B**) Panoramic views of the PCX layer II with EGFP^+^ cells (green) showing axon initial segments (AIS) (red) identified by the expression of the protein Ank-G. Note the presence of a larger number of EGFP^+^ complex cells displaying an AIS in the Endo-N hemisphere. (**B1**) Higher magnification of the squared area in B. An arrowhead indicates the AIS. (**C**) Graph showing a higher density of EGFP^+^ complex cells expressing Ank-G in the Endo-N hemisphere. Asterisks indicate statistically significant differences between hemispheres (Vehicle vs. Endo-N) after paired Student’s *t*-test. (* *p* ≤ 0.05) Scale bar represents 30 µm for A and B, and 55 µm for B1.

The density of EGFP^+^ cells displaying an AIS was investigated based on detecting the structural protein Ank-G to scrutinize whether PSA depletion could promote the differentiation of complex cells (Figure 5A,B). Interestingly, a significantly higher density of EGFP^+^ complex cells bearing an Ank-G-labeled AIS was detected in the Endo-N-treated hemisphere (*p* = 0.04, Figure 5C). Ank-G-labeled AIS were not observed on tangled cells.

### 2.5. Effects of PSA Depletion on EGFP^+^ Dendrites and Dendritic Spines in the PCX Layer I

**Figure 6:**
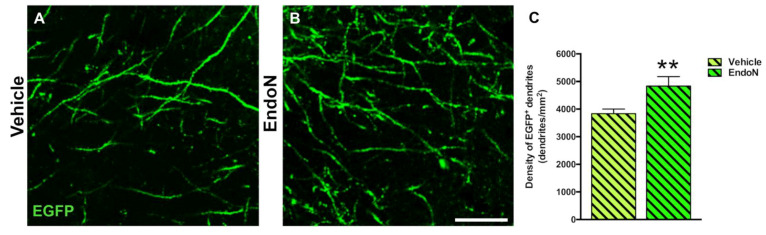
Confocal analysis of the density of EGFP^+^ dendrites in the PCX layer I. (**A**,**B**) Single confocal planes showing dendritic processes in the vehicle (**A**) and Endo-N (**B**) hemispheres. (**C**) Graph showing a higher density of dendrites in the Endo-N versus the vehicle hemisphere. Asterisks indicate statistically significant differences between hemispheres (Vehicle vs. Endo-N) after paired Student’s *t*-test. (** *p* < 0.01). Scale bar: 10 µm.

**Figure 7:**
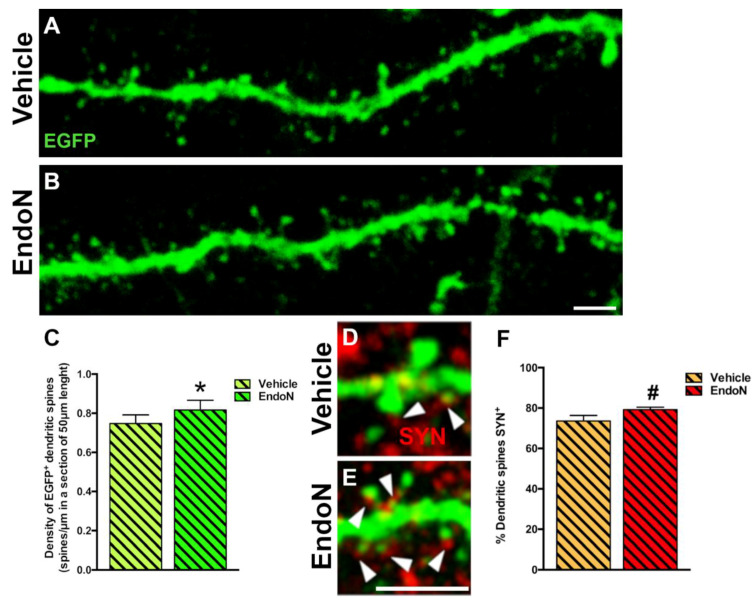
Confocal analysis of the density of dendritic spines in the PCX layer I. (**A**,**B**) 2D projections of confocal stacks (20 confocal planes separated by 0.38 µm) of spiny dendrites from the vehicle (**A**) and Endo-N (**B**) hemispheres. (**C**) Graph showing increased the density of dendritic spines in a segment of 50 µm in the Endo-N hemisphere. (**D**,**E**) Higher magnification views of the dendritic spines of complex EGFP^+^ cells in close apposition to puncta expressing the presynaptic markers SYN (red) on their surface. Note the presence of a larger number of puncta expressing SYN (arrowheads) in the Endo-N hemisphere (**E**) when compared to the vehicle hemisphere (**D**). (**F**) Graphs showing the percentage of puncta expressing SYN apposed to EGFP^+^ spines. Asterisks indicate statistically significant differences between hemispheres (Vehicle vs. Endo-N) after paired Student’s *t*-test. (* *p* ≤ 0.05; 0.05 < ^#^ *p* < 0.1). Scale bar: 5 µm (**B**) and 15 µm (**E**).

To question whether developing dendrites and dendritic spines on EGFP^+^ complex cells in the PCX layer I was affected by PSA depletion, we analyzed their density and compared both hemispheres. PSA depletion resulted in a significantly higher density of EGFP^+^ dendrites in the PCX layer I of the Endo-N hemisphere (*p* = 0.001, Figure 6). Next, we analyzed the density of dendritic spines on the EGFP^+^ dendrites and observed a significant increase in the Endo-N hemisphere (*p* = 0.03, Figure 7A–C). However, analysis of the different spines subtype (mushroom, stubby and thin) revealed no shift of distribution between the various morphologies upon PSA depletion (Supplementary Materials Figure S2).

To explore the connectivity and degree of maturation of the dendritic spines of EGFP^+^ neurons, we also analyzed the presence of closely apposed puncta expressing the presynaptic marker SYN. We observed a slight increase in the frequency of spines apposed to SYN^+^ puncta following Endo-N treatment. However, this difference did not reach significance (Figure 7D–F).

### 2.6. PSA Depletion Did Not Change the Density of Excitatory and Inhibitory Synaptic Puncta in the Perisomatic Region of EGFP^+^ Complex Cells

**Figure 8:**
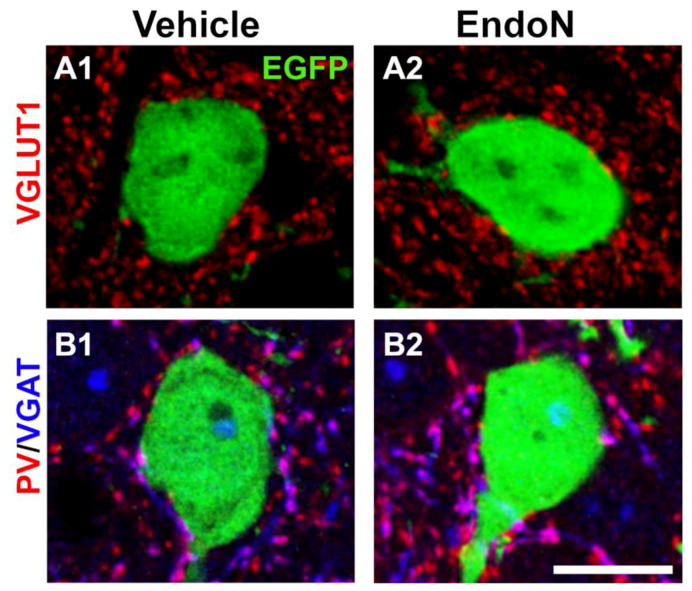
Excitatory and inhibitory puncta in the perisomatic region of EGFP^+^ complex cells in the PCX layer II. (**A**,**B**) 2D projections of single confocal stacks showing VGLUT1^+^ (**A**, red), VGAT^+^ (**B**, blue) and PV^+^ (**B**, red) puncta in the perisomatic region of EGFP^+^ complex cells (green) in the vehicle (**A1**,**B1**) and Endo-N (**A2**,**B2**) injected hemispheres. Scale bar: 10 µm.

To study whether EGFP+ complex cells receive synaptic inputs in their perisomatic region and to investigate the nature of these afferences, we analyzed the presence of puncta expressing VGLUT1 and VGAT, which are markers of glutamatergic and GABAergic terminals, respectively and of PV+ puncta belonging to basket cells (Figure 8A,B). In both hemispheres, we found numerous VGLUT1+, VGAT+ and PV+ puncta in the perisomatic region of EGFP+ complex cells. However, no significant differences were detected in the density of these 3 afferents when comparing the 2 hemispheres (Supplementary Materials Figure S3).

## 3. Discussion

In this work, we explored in a DCX-CreERT2/Flox-EGFP transgenic mouse model the impact of PSA depletion to understand its role in the quiescence and differentiation of immature neurons within the adult PCX layer II. To the best of our knowledge, these results demonstrate for the first time that an injection of the enzyme Endo-N in the ectorhinal cortex depletes the PSA from the mouse PCX for up to 2 weeks. We provided evidence that an enzymatic depletion of PSA promoted the final stages of development of immature neuronal populations, as demonstrated by the significant increase in the density of NeuN^+^ nuclei in the adult PCX layer II. We also observed a significant increase in the density of EGFP^+^ complex cells displaying an AIS. Moreover, we found higher densities of EGFP^+^ dendrites and dendritic spines within the PCX layer I. In agreement with previous reports, we did not observe evidence for Endo-N-induced neuronal death based on the absence of necrotic cells and pyknotic nuclei (data not shown) [11,13,28,29,37,38].

In this study, we classified EGFP^+^ cells in 5 different subcategories, reflecting their stages of maturation. In analogy with the development and functional integration of newborn dentate granule cells (DGCs) during adult neurogenesis [39], the process of maturation of EGFP^+^ cells occurs through several intermediate steps, involving a series of events that are essential for their functional maturation. The EGFP^+^ cells in the PCX layer II followed a similar pattern of morphological and functional maturation with immature cells expressing early neuronal markers. The latter get progressively down-regulated to make place for mature neuronal markers expression [5,18,35,40,41]. In the subgranular zone of the dentate gyrus, PSA-NCAM expression was also found on immature neurons and PSA depletion through the administration of Endo-N was reported to enhance neuronal differentiation [32].

In agreement with our expectations, PSA removal affected primarily the maturation of the tangled cells and early complex cell subpopulations in the PCX, i.e., the cells bearing the largest amount of PSA-NCAM on their membranes. Upon 2 weeks of PSA depletion, immature neurons induced more frequently the expression of NeuN than their vehicle-treated counterpart. The fact that the percentage of CAMK-II-expressing EGFP^+^ cells did not increase following PSA depletion suggests that immature neurons require more time to reach a fully differentiated phenotype than our short observation window.

The role of Endo-N as a promoter of developing EGFP^+^ cells is also demonstrated from structures related to physiological neuronal maturation, such as the increase in the density of dendritic branches and dendritic spines in PCX layer I. Analyses on both parameters suggested increased the synaptic input on EGFP^+^ immature neurons of the PSA-depleted hemisphere. Furthermore, Endo-N treatment promoted the presence of AIS, which is an electrogenic axonal domain essential for action potential initiation, on immature neurons [42,43,44]. We recently reported that the presence of AIS on mature, complex cells of the PCX layer II provided the capacity of repetitive action potential firing that was absent in less mature precursors devoid of AIS [15].

As the development of immature neurons progresses, in addition to morphological changes, there must be a parallel increase in connectivity. The latter is normally revealed by a combination of afferent input through dendrites and efferent output through axons. As described in previous studies, only EGFP^+^ complex cells that had undergone 6 months of maturation, and not the tangled cells, were surrounded by SYN and VGAT immunoreactive puncta and showed dendritic spines and expression of markers of glutamatergic synapses, indicating their integration into adult neuronal networks [11,15]. Importantly, the observation that the percentages of EGFP^+^ spines apposed to SYN^+^ presynaptic puncta and that the density of PV^+^, VGLUT1^+^ and VGAT^+^ perisomatic puncta were similar in both hemispheres indicated that the afferent input sources did not constitute a limiting factor during the accelerated integration of the late-maturing neurons in the PCX.

The current data and several previous studies [6,11,13,15] suggest that immature neurons in the PCX layer II and particularly the EGFP^+^ cells in the DCX-CreERT2/Flox-EGFP transgenic mouse, have an excitatory fate and are integrated into the circuitry as pyramidal neurons. Therefore, we decided to study the effect of PSA removal on the expression of CAMK-II, a marker for fully mature excitatory neurons [36]. However, 2 weeks after Endo-N treatment, no changes in the percentage of EGFP^+^ cells expressing CAMK-II in the adult PCX layer II were detected. We think that this lack of effect is due to the early time point of analysis following PSA depletion.

A different hypothesis can be postulated on the mechanisms by which PSA depletion may accelerate the differentiation of the most immature neurons in the PCX. First, PSA-NCAM may have an insulating role that prevents forming synaptic contacts on these immature neurons or affects the NCAM signaling [5]. In fact, different reports have demonstrated that the polysialylated form of NCAM, through its large hydration volume, decreased its homophilic bindings, which may influence the heterophilic interactions with other molecules and receptors [22,23,45]. Consequently, removing PSA from NCAM may facilitate specific signaling transduction pathways that may promote the final development of immature neurons. On the other hand, PSA-NCAM acts as an anti-adhesive molecule, which facilitates neurodevelopmental events. During the first stages of neuronal development, PSA-NCAM plays an important role in promoting cell migration, cell differentiation and also facilitates axonal outgrowth, neuronal-glial structural remodeling and synaptogenesis [22,23,24,25,46,47]. Hence, we cannot exclude that the depletion of PSA-NCAM negatively affects the very early steps of maturation of tangled cells. Dityatev et al. (2004) demonstrated that Endo-N treatment blocked synaptogenesis in primary neuronal cultures from the early postnatal hippocampus. It is also interesting to note that although the removal of PSA from NCAM accelerated the differentiation of neuronal progenitors in the SGZ, it blocked their subsequent migration to the upper layers of the granule cell layer [32]. Apart from the differences in the type and stage of development of the neuronal progenitors studied, we also believe that the different regional/temporal environments may also influence the effects of PSA-NCAM**.**

Our study highlights the important role of PSA-NCAM expression in maintaining the immature state of these neurons in the adult cerebral cortex. PSA-NCAM progressive downregulation allows the final differentiation of these cells, which can be precipitated with an enzymatic depletion of the PSA moiety. More study will be required to understand the intrinsic and extrinsic factors affecting PSA-NCAM expression in the immature cells of the PCX to understand how the brain regulates their incorporation to the established neuronal circuits. The functional role of these cells once incorporated into the PCX circuitry is still a mystery. Immature neurons do not appear to be a source for a neuronal replacement since no solid evidence of cell death has been found in this region. The actual lack of knowledge on their connectivity or their putative involvement in cognitive phenomena hinders understanding of their role in the adult brain. Taken that similar immature neurons populate most of the extension of the cerebral cortex of gyrencephalic species, including humans, complex cells may constitute a crucial resource for cortical plasticity [7,8,9,10,11,12]**.**

## 4. Material and Methods

### 4.1. Transgenic Animals

Fourteen young-adult transgenic DCX-CreERT2/Flox-EGFP mice [33] were used for studying neuronal maturation in the PCX after PSA depletion by Endo-N. In this transgenic strain, the expression of the EGFP reporter gene in DCX-expressing cells was induced by tamoxifen administration (100 mg/kg body weight (bw) dissolved in corn oil, Sigma-Aldrich, St. Louis, MO, USA). Tamoxifen was administered orally by gavage for 5 consecutive days in mice at 2 months of age.

Experiments were performed in agreement with the “Directive 2016/63/EU of the European Parliament and of the Council of 22 September 2010 on the protection of animals used for scientific purposes” and were approved by Austrian animal care authorities: protocol number: BMBWF-66.019/0025-V/3b/2019.

### 4.2. Polysialic Acid Depletion

Two days after the last injection of tamoxifen, mice were anesthetized with ketamine (75 mg/kg bw, i.p.) and medetomidine (1.5 mg/kg bw, i.p.) and afterward received carprofen (5 mg/kg bw s.c.). Mice were then placed in a stereotaxic instrument (David Kopf Instruments) and received an intracranial injection of either 1 µL of Endo-N (0.7 U/µL in glycerol; AbCys, France), using a Hamilton syringe, in the right hemisphere or of vehicle solution (saline and glycerol 1:1) in the left hemisphere. Endo-N is a phage enzyme that specifically cleaves α-2,8-linked N-acetylneuraminic acid polymers with a minimum chain length of 8. Endo-N diffuses rapidly and removes all local PSA within 1 day, and depletion remains for 3 to 4 weeks [48]. Since Endo-N can diffuse long-distances in the brain parenchyma, the enzyme was injected in the ectorhinal cortex (Bregma coordinates: 1.58 mm; lateral ± 4 mm; deep −3.4 mm) [34] to avoid lesion or edema in the PCX through the intracerebral injection. Endo-N (1 µL) was slowly injected in the right hemisphere over 4 min, and the needle was left in place after injection for an additional minute to avoid reflux into the needle track. Next, the vehicle solution was injected into the left hemisphere following the same procedure. After surgery, the mice received atipamezole (5 mg/kg bw, i.p.), and enrofloxacin (10 mg/kg bw, s.c.) was also administered on the day of surgery until the fifth day after post-operation to prevent the occurrence of infection. Recovery and wellbeing of mice after surgery were secured by careful postoperative care management.

### 4.3. Histological Procedures

Two weeks after surgery, mice were deeply anesthetized by intraperitoneal injection of ketamine (205 mg/kg bw), xylazine (53.6 mg/kg bw) and acepromazine (2.7 mg/kg bw). Mice were then transcardially perfused, first for 1 min with NaCl 0.9% and then for 30 min with 4% paraformaldehyde (PFA, Sigma-Aldrich, St.Louis, MO, USA) in phosphate buffer (PB) 0.1 M, pH 7.4. Thirty minutes after perfusion, brains were extracted from the skull, postfixed in PFA solution for 24 h. Then, brains were washed in phosphate saline buffer (PBS), and separated hemispheres were sliced coronally with a VT1200s microtome (Leica, Heidelberg, Germany) at a thickness of 50 µm. Sections were collected in 6 subseries and stored in 0.1 M PBS with sodium azide 0.05% as a preservative at 4 °C until used.

### 4.4. Immunohistochemistry

Tissue sections were processed free-floating as follows: briefly, sections were incubated for 1 min in an antigen unmasking solution (0.01 M citrate buffer, pH 6) at 100 °C. After cooling to room temperature, sections were incubated with 10% normal donkey serum (NDS; Sigma-Aldrich, St. Louis, MO, USA) in PBS with 0.2% Triton-X 100 (PBST; Sigma-Aldrich, St. Louis, MO, USA) for 1 h, before 48 h of incubation at 4 °C with the primary antibodies (Table 1). After washing, sections were incubated for 2 h at room temperature with the appropriate fluorescent conjugated secondary antibodies (Table 2). Nuclei were counterstained with DAPI (0.5 µg/mL; Sigma-Aldrich, St. Louis, MO, USA). Finally, sections were mounted on slides and coverslipped using Dako mounting medium (Agilent Technologies, Santa Clara, CA, USA).

### 4.5. Confocal Analysis and Quantification

Fluorescence images were acquired using an LSM 710 confocal microscope with ZEN 2011 Black software (Carl Zeiss, Cambrige, UK) and a TCS-SPE confocal microscope (Leica Mycrosystems, Heidelberg, Germany). Images were analyzed using the FIJI/ImageJ software; Bethesda, MD, USA [49].

#### 4.5.1. Classification and Phenotypic Characterization of EGFP Neurons

EGFP-expressing neurons were divided into 5 subgroups according to their soma diameter and maturational stage: tangled a: small, tangled cells (ø ≤ 5 µm) tangled b: tangled cells with short dendritic arborizations (ø = 6–7 µm); tangled c: tangled cells with longer dendritic arborizations (ø = 8–10 µm); complex d: larger complex cells with thin basal processes and dendritic spines (ø = 11–13 µm) complex e: mature, complex cells with larger dendrites and mature dendritic spines (ø ≥ 14 µm). To calculate the distribution in each subtype, 50 EGFP^+^ cells in the PCX layer II (Bregma interval: −1.58 mm; −2.54 mm) were randomly selected, and their morphology was analyzed. In addition, the frequency of EGFP^+^ cells immunolabeled with anti-NeuN or anti-CAMK-II antibodies was evaluated.

#### 4.5.2. Quantification of Cellular Densities

To perform analyses of nuclear and cellular densities, 4–7 sections per mouse were selected between the following coordinates: Bregma −1.58 mm to −2.54 mm. Three micrographs from a single confocal plane covering the extension of the PCX layer II were acquired using a 20× dry objective (Leica, Heidelberg, Germany) The density of NeuN-expressing cells and CAMK-II-expressing cells were measured within the PCX layer II using 15 randomly located regions of interest (ROI) per section (50 × 50 µm), and measurements were expressed as the number of structures per mm^2^.

#### 4.5.3. Quantification of Axonal Initial Segment Densities

To study whether the injection of Endo-N could accelerate the maturation of EGFP^+^ cells in the PCX layer II, we calculated the density of complex cells displaying an axon initial segment (AIS). For this purpose, we used an antibody directed against ankyrin-G (Ank-G) and the analysis was performed in a single ROI (single confocal plane, 200 × 200 µm) of a 1 section randomly selected from the Bregma interval: −1.58 mm; −2.54 mm.

#### 4.5.4. Analysis of Dendrites and Dendritic Spines

To analyze the density of EGFP^+^ dendrites in the PCX layer I, a single confocal plane was obtained using a 63× oil immersion objective (Leica, Heidelberg, Germany). The density of dendrites was assessed in a single section per mouse (Bregma interval: −1.58 mm; −2.54 mm) using 3 randomly located ROIs (50 × 50 µm). Measured densities were expressed as the number of dendrites per mm^2^.

We also studied the density of spines on the dendrites of EGFP^+^ neurons in the PCX layer I. Six dendrites were randomly selected from each mouse. To be suitable for dendritic spine analysis, the EGFP^+^ neurons bearing the selected dendrites had to fulfill the following criteria: (1) the soma should be located in the PCX layer II; (2) the dendrites should have a thickness >1 µm; (3) no other dendrites should be crossing their trajectory. A 63× oil immersion objective (Leica, Heidelberg, Germany) with a 3.5× additional digital zoom was used to analyze confocal stacks (Z-step size = 0.38 µm). The spines were quantified in a longitudinal dendritic 3D segment of 50 µm length, selected at a constant distance of 50–60 µm from the pial surface. The analysis also addressed the different types of dendritic spines and classified them according to the length of the protrusion and the diameters of their head and neck. Three different categories were established as described before [30]: (1) stubby, when the length of the protrusion was <1.5 µm; (2) mushroom, when a clear head could be observed (maximum diameter of the head should be at least 1.5 times the average length of the neck) and the total length of protrusion was <3 µm; and (3) thin, when the length of the protrusion was >3 µm or when this length was between 1.5 and 3 µm and a clear head could not be distinguished. Overall spine density was expressed as the number of spines/µm lengths.

#### 4.5.5. Analysis of Presynaptic Inputs on EGFP^+^ Spines

The presence of synaptophysin (SYN) in puncta closely apposed (distance: ≤3 µm) to 50 EGFP^+^ spines per mouse was scrutinized to analyze presynaptic inputs on EGFP^+^ spines. Results were expressed as the percentage of EGFP^+^ spines closely apposed to SYN expressing puncta.

#### 4.5.6. Analysis of Perisomatic Puncta Expressing Excitatory/Inhibitory Synaptic Markers

We studied the density of perisomatic puncta expressing VGLUT1, VGAT and PV on EGFP^+^ complex cells (ø ≥ 10.5 µm). Between 10–15 neurons were analyzed per mouse, and the analyses were performed in 2 randomly selected sections corresponding to Bregma interval: −1.58 mm; −2.54 mm. Confocal z-stacks covering the whole depth of the section were taken with 0.38 µm step-size, from which a single plane having an optimal penetration level for each antibody was selected. Images were processed using a customized macro for (FIJI/ImageJ software [50]. The soma profile of the complex neurons was drawn manually, and then the selection was enlarged by 0.5 µm to define an ROI along the perimeter of the soma. In this region, we analyzed the density of immunoreactive puncta having an area of at least 0.15 µm^2^ for VGLUT1 and VGAT and 0.30 µm^2^ for PV (circularity between 0.30 and 1.00). The data were expressed as immunoreactive puncta per µm of the soma perimeter.

## 5. Statistical Analysis

Analyses were performed comparing the right hemisphere (Endo-N) with the left hemisphere (vehicle). To avoid bias in the analyses, the slides were coded, and analyses were performed in a blinded manner until the end of data acquisition. Statistical analyses were performed using GraphPad Prism 9 software (GraphPad Software Inc.; La Jolla, CA, USA). After assessing the normality of the data via Shapiro–Wilk test, a 2-tailed paired Student’s t-test was applied. Statistical significance was assumed for *p* ≤ 0.05. Data are shown as mean ± standard error of the mean (SEM).

## Figures and Tables

**Table 1:** Primary antibodies.

Anti	Host	Isotype	Dilution	Cat Number	Company
Ank-G	Mouse	IgG_1_	1:200	SC12719	Santa Cruz
CAMK-II	Mouse	IgG_1_	1:1000	ab22609	Abcam
DCX	Rabbit	IgG	1:2000	ab207175	Abcam
GFP	Chicken	IgY	1:1000	A10262	Thermo Fisher
NeuN	Mouse	IgG_1_	1:1000	MAB377	Sigma-Aldrich
PSA-NCAM	Mouse	IgM	1:1400	MAB5324	Sigma-Aldrich
PV	Guinea pig	IgG	1:2000	195004	SySy
SYN	Mouse	IgG_1_	1:1000	S5768	Sigma-Aldrich
VGAT	Rabbit	IgG	1:1000	131002	SySy
VGLUT1	Guinea pig	IgG	1:1000	AB5905	Sigma-Aldrich

**Table 2:** Secondary antibodies.

Anti	Host	Conjugate	Dilution	Cat number	Company
Chicken IgY	Donkey	CF488	1:400	20020	Biotium
Guinea pig IgG	Goat	AF647	1:400	106605003	Jackson ImmunoResearch
Mouse IgG_1_	Goat	CF647	1:400	20252	Biotium
Mouse IgG_1_	Goat	AF555	1:400	A21127	Thermo Fisher
Mouse IgM	Goat	CF555	1:400	20485	Biotium
Rabbit IgG	Donkey	CF555	1:400	20038	Biotium

## Data Availability

The data presented in this study are available on request from the corresponding authors.

## Supplementary Materials

The following are available online at https://www.mdpi.com/article/10.3390/ijms22115733/s1.
