# Supplementary figures and images for: PSA Depletion Induces the Differentiation of Immature Neurons in the Piriform Cortex of Adult Mice
Source: Int J Mol Sci. 2021 May 27;22(11):5733. doi: 10.3390/ijms22115733 (PMC8198564; doi:10.3390/ijms22115733)

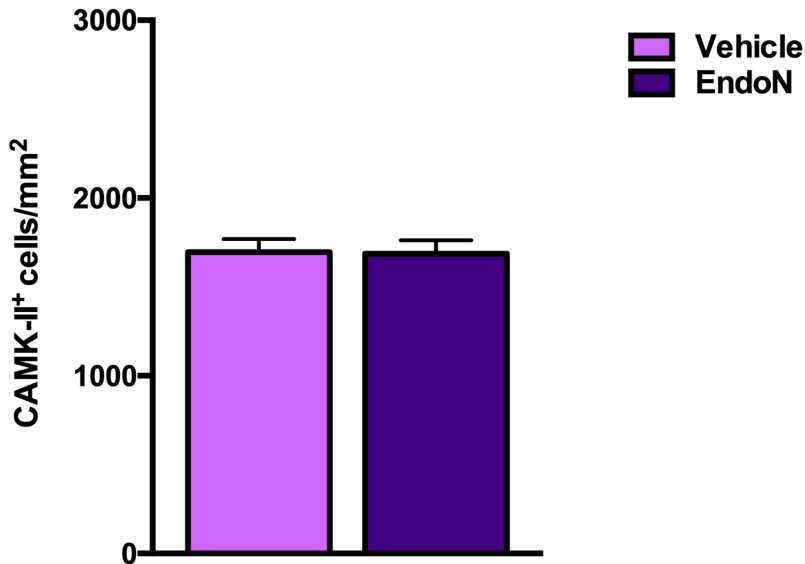

Supplement: Supplementary file 1 [file ijms-22-05733-s001.zip › Supplementary Figure 1.pdf]

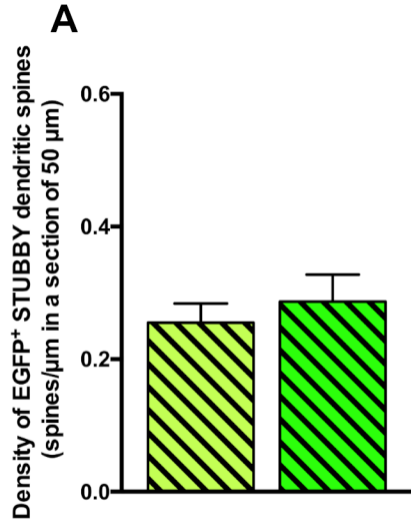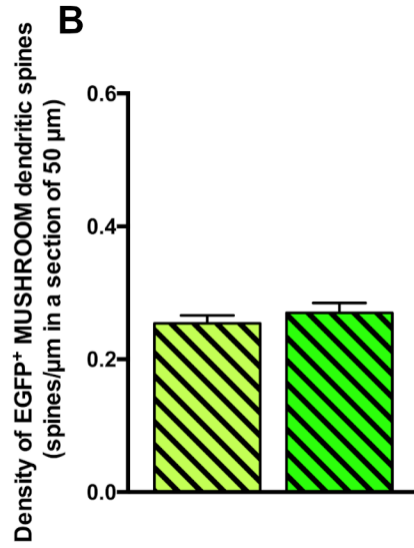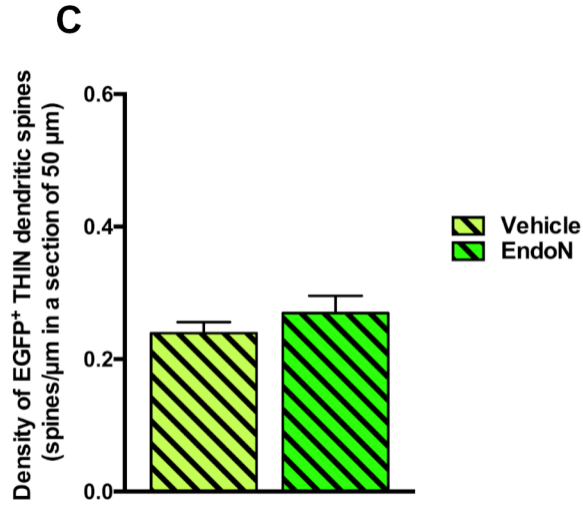

Supplement: Supplementary file 1 [file ijms-22-05733-s001.zip › Supplementary Figure 2.pdf]

**A**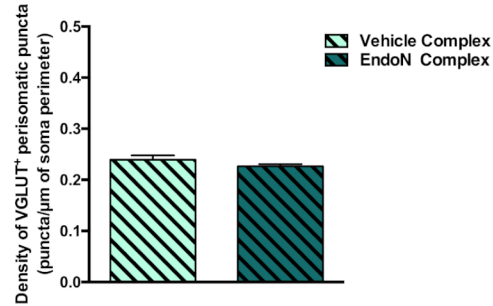**B**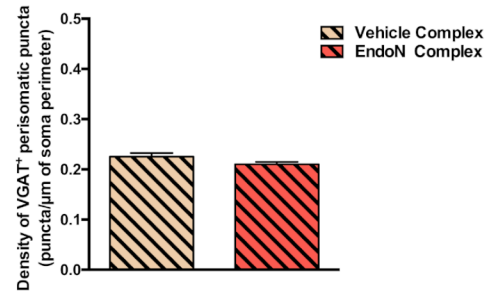**C**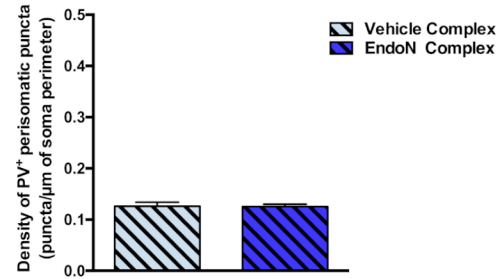**D**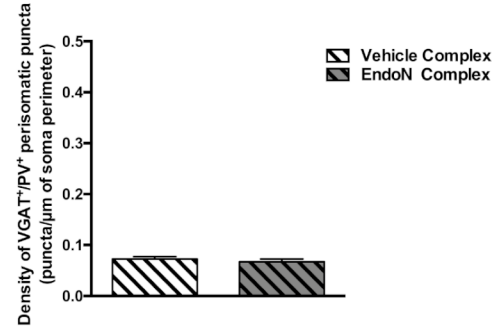

Supplement: Supplementary file 1 [file ijms-22-05733-s001.zip › Supplementary Figure 3.pdf]
